# Beyond Genetic Conservation: The Baton Pass Model of Essential Biological Functions

**DOI:** 10.3390/biom16060894

**Published:** 2026-06-17

**Authors:** Takayuki Miyazawa

**Affiliations:** Kyoto Animal-Human-Organism Research Institute (Kyoto AHOI), 390-2 Shimomaruyacho, Nakagyo-ku, Kyoto 604-8006, Japan; takavet@ahoi.or.jp

**Keywords:** Baton Pass model, endogenous retrovirus, syncytin, placentation, receptor conflict, host–virus co-evolution, functional turnover

## Abstract

Essential host functions are often maintained by conserved molecular systems, but in biological contexts shaped by evolutionary conflict, the genes that execute such functions may be unstable, replaceable, or repeatedly recruited from different evolutionary sources. Mammalian placentation provides a striking example of this principle. Trophoblast cell fusion is essential for placental development, yet this function is mediated in different mammalian lineages by distinct endogenous retrovirus-derived envelope proteins, including syncytin-1, syncytin-2, and other lineage-specific Env-derived fusogens. Here, I propose the Baton Pass model as a conceptual framework for explaining how host-level biological functions can be maintained despite turnover of the molecular agents that execute them. This model differs from conventional examples of antagonistic coevolution, which often emphasize recurrent mutations within the same interacting genes, and from non-orthologous gene displacement, which generally concerns replacement among cellular genes. In the syncytin paradigm, the molecular executors are repeatedly supplied by exogenous retroviral env genes that become endogenized, domesticated, and incorporated into host developmental programs. I further discuss how receptor compatibility, placental expression control, and host–virus evolutionary conflict may together destabilize individual Env–receptor systems while allowing the host-level function of trophoblast fusion to persist. Analogous functional reassignment is also observed in primate lentiviruses, where antagonism of BST-2 shifts among distinct viral genes. The Baton Pass model therefore describes a testable evolutionary principle: essential host functions can be preserved not only through conservation of specific genes, but also through dynamic succession of genes of distinct evolutionary origins.

## 1. Introduction

### 1.1. Functional Conservation, Gene Conservation, and Evolutionary Conflict

Many essential biological functions are maintained by genes that are conserved over long evolutionary periods. In such cases, strong purifying selection preserves the integrity of genes required for survival, reproduction, development, or fundamental cellular processes. This principle explains the deep conservation of many core metabolic enzymes, developmental regulators, and components of basic cellular machinery. However, gene conservation is not the only possible route to functional conservation. In some biological systems, especially those shaped by persistent evolutionary conflict, the molecular agents that execute a function may be unstable, replaceable, or repeatedly recruited from different evolutionary sources.

The Red Queen hypothesis and theories of antagonistic coevolution provide a powerful framework for understanding rapid molecular change in biological systems shaped by conflict, including host–virus interactions and maternal–fetal interactions [1,2,3]. In many such cases, evolutionary instability is manifested as recurrent amino acid substitutions within the same interacting genes, such as viral entry proteins and host receptors or viral antagonists and host restriction factors. However, functional continuity can also be achieved by a more radical process: replacement of the molecular executor itself. The Baton Pass model focuses on this latter mode of evolution, in which a host-level biological function is preserved while the gene responsible for executing that function is replaced by another gene of distinct evolutionary origin.

In the syncytin paradigm, this replacement is especially distinctive because the substitute genes are not simply alternative host genes. Rather, they are derived from exogenous retroviral env genes that were independently acquired, endogenized, domesticated, and placed under host regulatory control. Thus, the Baton Pass model resembles non-orthologous gene displacement in that the same biological function can be executed by genes of different origins. However, it differs in emphasizing recurrent recruitment of foreign viral genes into host developmental programs, rather than replacement among pre-existing cellular genes.

### 1.2. Functional Turnover in Placentation

Mammalian placentation provides one of the clearest examples in which host-level functional conservation is achieved despite turnover of the genes that execute the function. In this review, the term “essential biological function” refers primarily to host-level functions that contribute to reproduction, development, or antiviral defense. This does not imply that the same molecular activity is beneficial to all biological entities involved. For example, the fusogenic activity of a retroviral Env protein originally serves viral entry, but after endogenization and domestication, the same activity can be repurposed for trophoblast fusion in the host placenta. Thus, the Baton Pass model explicitly distinguishes between the original viral function of a molecule and the host-level function that it may later execute. The Baton Pass hypothesis was originally proposed to explain successive functional replacement of ERV-derived fusogenic genes during mammalian placental evolution by Imakawa, Nakagawa, and Miyazawa [4], and further discussed in a subsequent review [5]. The Baton Pass hypothesis is extended here into a mechanistic framework, termed the Baton Pass model. In many mammals, successful placental development depends critically on trophoblast cell–cell fusion (in Bovidae, cell fusion between trophoblasts and maternal endometrial cells), a process required for the formation of syncytial structures and for efficient maternal–fetal exchange [6,7,8,9]. Disruption of this process is incompatible with normal embryonic development, underscoring its essential nature. Trophoblast cell fusion is mediated by envelope (Env) proteins derived from endogenous retroviruses (ERVs) [6,8,9,10,11,12,13]. These ERV-derived genes, collectively referred to as syncytins and related fusogens, retain hallmark features of viral envelope proteins, including receptor binding and membrane fusion activity. Comparative analyses indicate that some of these genes show signatures of purifying selection and lineage-specific functional specialization, consistent with important host roles.

At first glance, however, the genes associated with this essential process often exhibit lineage-specific attenuation, truncation, or loss [10,11,13,14]. Even among closely related species, an Env-derived gene that is intact and fusogenic in one lineage may be partially degraded or nonfunctional in another, while trophoblast fusion as a biological process is preserved. Traditional explanations such as genetic redundancy or relaxed selection do not fully account for this phenomenon. Redundancy would predict the stable coexistence of multiple fully functional genes, whereas many genomes instead show rapid decay of individual Env-derived genes. Likewise, relaxed selection is difficult to reconcile with the persistence of a function that is demonstrably indispensable. These observations raise a central question: how can evolution tolerate repeated loss of genes associated with essential functions, while preserving the underlying biological process?

Furthermore, recent comprehensive reviews highlight that mammalian placentation has been shaped by multiple, independent exaptation events of diverse ERV genes, extending beyond fusogens to include elements like suppressyn that modulate or inhibit cell fusion [15,16].

**Figure 1:**
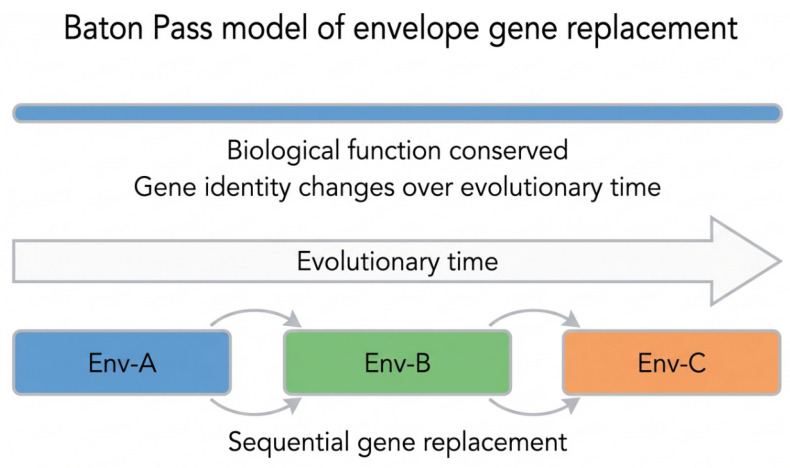
Conceptual model of Baton Passing in ERV-derived envelope genes. A conserved host-level function is maintained over evolutionary time, whereas the Env-derived genes that execute the function are sequentially replaced. Env-A, Env-B, and Env-C each contribute to the function during limited evolutionary intervals and are subsequently lost, attenuated, or replaced. This process allows functional continuity despite turnover of the responsible genetic elements.

This review was motivated by comparative studies of ERV-derived envelope genes involved in placental fusion, particularly syncytin-1 in primates and ruminant Env genes such as fematrin-1 and syncytin-Rum1. These genes represent independent domestication events of retroviral envelope proteins that perform analogous fusogenic roles in distinct mammalian lineages. They suggest that specific Env genes are not universally indispensable, even though trophoblast fusion remains a conserved host-level requirement. Building on this insight, I propose the Baton Pass model as a framework for understanding how placental fusion can persist despite recurrent turnover of the genes that execute it (Figure 1).

## 2. Conceptual Framework and Predictions of the Baton Pass Model

### 2.1. Definition and Core Components

The Baton Pass model explains how a host-level biological function can be maintained despite turnover of the genes that execute it. In this model, natural selection can preserve the biological outcome of a function while allowing replacement of the molecular agents responsible for that outcome. In the case of ERV-derived envelope genes, Baton Passing refers to a process in which the fusogenic activity required for placental development is carried by one Env gene for an evolutionarily limited period and is subsequently reassigned to another Env-derived candidate.

This process differs from simple redundancy. At any given evolutionary stage, one or a small number of Env genes may be functionally engaged, whereas other related sequences remain inactive, partially functional, or latent within the genomic reservoir. The loss or degradation of a previously functional Env gene therefore does not necessarily imply loss of the biological function itself. Instead, it may indicate that the function has been reassigned to another molecular executor.

The model has three core components. First, mammalian genomes contain endogenous retroviral reservoirs, including numerous env-derived sequences at different stages of degradation, some of which may retain structural integrity, expression potential, or partial fusogenic activity. Second, Env–receptor interactions are inherently unstable because host receptors can be shaped by antiviral selection and host–virus co-evolution. Such receptor conflict may reduce the functional lifespan of individual Env–receptor combinations and create opportunities for replacement. Third, ERV-derived Env genes are expected to experience episodic rather than continuous purifying selection: they may be constrained while they execute a host function, but may decay after that role is transferred to another gene.

Thus, Baton Passing decouples functional continuity from long-term conservation of a specific gene. The “baton” is not simply a DNA sequence, but a functional role that can be carried by different molecular agents over evolutionary time. In the syncytin paradigm, this role includes not only fusogenic coding capacity, but also compatibility with a cellular receptor and integration into placental regulatory programs.

### 2.2. Testable Predictions

The Baton Pass model generates several testable predictions. First, related species may preserve comparable host-level functions while relying on different Env-derived genes, resulting in lineage-specific gene loss without loss of the biological outcome. Second, individual Env genes should show episodic signatures of purifying selection, with periods of functional constraint followed by relaxation and decay after functional replacement. Third, changes in host receptors or regulatory contexts may correlate with the attenuation of existing Env genes and the recruitment of alternative candidates. Fourth, successful replacement should involve not only coding sequences, but also regulatory compatibility, including placental or trophoblast-specific expression.

These predictions distinguish Baton Passing from simple redundancy, in which multiple functional genes are maintained in parallel, and from ordinary gene decay, in which loss of a gene reflects loss of functional relevance. In the Baton Pass model, gene decay can occur after the biological role has been transferred to another molecular agent.

## 3. Evidence for Baton Passing in Host Genomes

### 3.1. Comparative Genomics and Functional Attenuation of ERV-Derived Env Genes

Large-scale comparative genomic analyses of endogenous retrovirus-derived sequences provide the first line of evidence consistent with the Baton Pass model [17,18,19]. Across mammalian genomes, ERV-derived env genes exhibit a heterogeneous evolutionary landscape. Most env-derived sequences have accumulated frameshifts, premature stop codons, or deletions, consistent with neutral decay. However, a limited subset retains long open reading frames, fusogenic potential, or signatures of purifying selection, suggesting that some env genes have been recruited for host functions.

Importantly, the identity of these preserved genes varies among mammalian lineages. An env-derived gene that appears functionally constrained in one clade may be truncated or attenuated in another, while the host-level process of placental fusion is maintained. This lineage-specific dissociation between gene integrity and biological outcome is difficult to explain by simple long-term conservation of a single essential gene, but is consistent with sequential functional replacement.

Primate syncytins illustrate this pattern. Syncytin-1 is functionally constrained and fusogenic in hominoids, whereas related or orthologous sequences in other primate lineages may show reduced activity, altered processing, or partial disruption [8,13]. Similarly, syncytin-2 shows lineage-dependent variation in coding integrity and fusogenic activity, including reduced activity or partial open reading frame disruption in some New World monkeys [14]. These observations suggest that no single env-derived gene is universally required across all primates, even though trophoblast fusion remains a conserved biological requirement.

**Figure 2:**
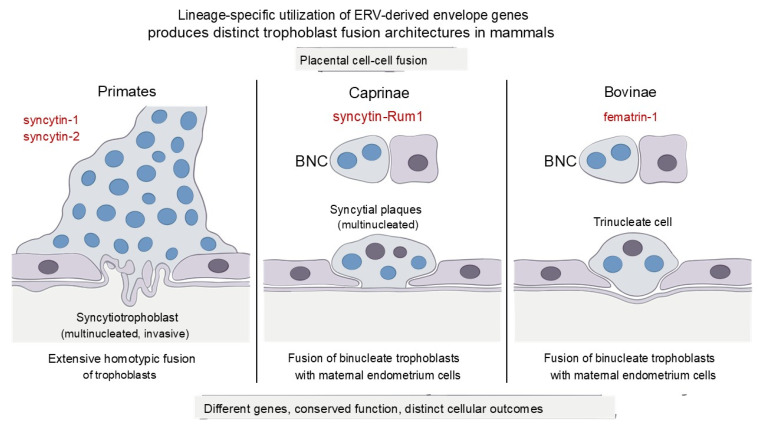
Lineage-specific ERV-derived envelope genes generate distinct trophoblast fusion architectures. In primates, syncytin-1 and syncytin-2 mediate trophoblast–trophoblast fusion, forming a multinucleated syncytiotrophoblast layer. In Caprinae, syncytin-Rum1 contributes to fusion between trophoblast binucleate cells and maternal epithelial cells, generating syncytial plaques. In Bovinae, fematrin-1 promotes formation of trinucleate fetomaternal hybrid cells. These examples illustrate that a conserved host-level function—placental cell fusion—can be achieved by lineage-specific env-derived genes and distinct cellular mechanisms, consistent with the Baton Pass model.

Ruminant placentation provides an independent example. Distinct ERV-derived env genes, including syncytin-Rum1 in Caprinae and fematrin-1 in Bovinae, contribute to placental cell fusion in a lineage-dependent manner [7,9,20]. These systems differ not only in gene identity, but also in the cellular architecture produced by trophoblast fusion. In Caprinae, trophoblast binucleate cells form syncytial plaques with maternal epithelial cells, whereas in Bovinae, fematrin-1 contributes to the formation of trinucleate fetomaternal hybrid cells. Thus, a conserved host-level function is achieved through lineage-specific molecular and cellular mechanisms (Figure 2).

An alternative, not mutually exclusive explanation is that ancestral or early-diverging placental mammals possessed a broader repertoire of fusogenic ERV-derived env genes, from which different lineages selected distinct candidates during diversification. Under this view, syncytin succession may reflect both historical availability of multiple env candidates and lineage-specific retention of those compatible with local receptor usage and placental regulatory programs. The receptor-compatibility model proposed here should therefore be understood as one mechanism that can bias the retention or loss of candidate env genes, rather than as the sole explanation for syncytin turnover.

Together, these examples support a central prediction of the Baton Pass model: functional continuity can be maintained even when the specific ERV-derived env genes that execute the function differ among lineages. This pattern is more consistent with temporal recruitment and replacement than with stable preservation of a single universally conserved fusogen.

Recent genomic evidence further supports this concept of progressive exaptation in Bovinae, illustrating how sequential recruitment of different ERV elements has driven the structural diversity and evolution of ruminant placentation [21].

### 3.2. Regulatory Compatibility: Placenta-Specific Expression of Syncytins

Receptor compatibility alone is not sufficient for a retroviral Env protein to become a functional syncytin. A fusogenic Env must also be expressed in the appropriate tissue, developmental stage, and cell type, while potentially deleterious expression in non-placental tissues must be suppressed. Thus, domestication of an env gene requires not only preservation of fusogenic activity and compatibility with a cellular receptor, but also incorporation into host regulatory networks that drive placental or trophoblast-specific expression. This requirement can be viewed as regulatory compatibility, which acts together with receptor compatibility to determine whether an endogenous retroviral env gene can be retained as a functional syncytin.

Evidence for functional turnover is not limited to protein-coding sequences but extends to regulatory elements required for gene expression. Functional co-option of ERV-derived envelope genes depends not only on fusogenic activity but also on appropriate transcriptional regulation, RNA processing, and translation. One example is the syncytin post-transcriptional regulatory element (SPRE), which enhances expression of syncytin-1 and syncytin-2 [22]. SPRE-like elements are found in multiple independently acquired ERV-derived genes, suggesting that regulatory modules can also be retained or reassembled during functional recruitment. At the same time, comparative analyses indicate that coding sequences and regulatory elements can become decoupled. In some cases, Env open reading frames are preserved while associated regulatory elements are degraded, potentially limiting functional expression. Conversely, newly recruited Env genes may acquire or evolve compatible regulatory features. These observations indicate that functional recruitment of syncytins cannot be inferred from the integrity of the env open reading frame alone. A newly acquired env gene must also retain or acquire regulatory elements that permit sufficient expression in trophoblast cells, whereas an env gene that preserves fusogenic coding capacity may become functionally irrelevant if its regulatory context is lost, silenced, or disconnected from placental expression programs.

Regulatory compatibility also includes transcriptional control at the promoter and enhancer levels. Recent epigenomic studies indicate that transposable elements, including ERV-derived sequences, can function as promoters or enhancers that shape trophoblast-specific gene expression programs [23,24]. Such regulatory elements may help integrate newly acquired viral-derived genes into placental transcriptional networks. Therefore, Baton Passing should not be understood as replacement of coding sequences alone, but as replacement and reassembly of coding and regulatory modules that together generate a functional placental phenotype.

Epigenetic regulation provides another layer of control. For example, syncytin-1 expression is associated with regulatory features of the ERVWE1 5′LTR, including DNA methylation status, and altered methylation has been linked to changes in placental syncytin-1 expression [25,26]. Such mechanisms are important not only for activating env-derived genes in trophoblast cells, but also for preventing inappropriate expression in non-placental tissues. In this sense, domestication requires both positive regulation in the placenta and negative regulation elsewhere.

The functional co-option of ERV-derived genes necessitates precise spatiotemporal regulation integrated into host networks. A prime example is Suppressyn, whose placenta-specific expression is tightly controlled not only by DNA methylation but also by novel internal enhancer elements and hypoxia-inducible factors (HIFs) in response to oxygen concentrations [27,28].

These considerations refine the Baton Pass model. Functional succession of syncytins requires more than the replacement of one fusogenic Env protein by another. It requires the emergence of a complete functional unit consisting of an env coding sequence, a compatible receptor interaction, and a regulatory context that ensures appropriate placental expression. Thus, the baton that is passed during evolution is not merely a protein-coding gene, but a functional module embedded in host developmental regulation.

### 3.3. Functional Continuity Through Molecular Turnover

The evidence summarized above supports a model in which ERV-derived env genes undergo functional turnover rather than long-term permanence. Comparative genomic analyses reveal lineage-specific retention, attenuation, and loss of candidate fusogens, whereas phenotypic observations suggest that placental fusion is maintained despite variation in gene identity. Regulatory analyses further indicate that functional recruitment requires not only fusogenic coding capacity, but also integration into placental expression programs.

These patterns are difficult to explain solely by redundancy or relaxed selection. Instead, they suggest that ERV-derived env genes can act as temporally limited carriers of a host-level function. In this framework, decay of an individual env gene does not necessarily indicate loss of the biological function, but may reflect functional succession to another env-derived gene or regulatory module.

## 4. Distinction from Related Evolutionary Concepts

### 4.1. Redundancy and Convergent Evolution

The Baton Pass model should be distinguished from both genetic redundancy and convergent evolution. Genetic redundancy refers to the simultaneous presence of multiple genes capable of performing similar functions. In such cases, functional robustness is achieved by parallel backup systems, and loss of one gene may have little phenotypic effect because another functionally overlapping gene remains active. By contrast, Baton Passing emphasizes temporal succession rather than stable coexistence. Although transient overlap between Env-derived genes may occur, the model does not require long-term maintenance of multiple fully functional fusogens in parallel. Instead, a host-level function can be preserved as the molecular executor is replaced over evolutionary time.

Baton Passing also differs from classical convergent evolution. Convergent evolution describes the independent emergence of similar traits in distinct lineages under similar selective pressures. In contrast, the Baton Pass model emphasizes functional continuity through sequential recruitment from a pre-existing or newly acquired repertoire of ERV-derived genes. In the syncytin paradigm, different lineages may use distinct env-derived genes and generate different cellular architectures, yet the host-level function of placental cell fusion is maintained. Thus, Baton Passing combines functional continuity with molecular discontinuity, rather than simply describing independent origins of similar traits.

### 4.2. Non-Orthologous Gene Displacement and the Unique Features of Baton Passing

The Baton Pass model also resembles non-orthologous gene displacement in that the same biological function can be executed by genes of different evolutionary origins. However, the two concepts are not identical. Non-orthologous gene displacement generally refers to cases in which a cellular function is performed by unrelated or distantly related genes in different organisms or lineages [29]. It is often discussed in the context of replacement among cellular genes or genes acquired through horizontal transfer.

The syncytin paradigm differs in a crucial respect. The replacing genes are not merely alternative host genes. Rather, they are derived from exogenous retroviral env genes that were independently acquired, endogenized, domesticated, and incorporated into host developmental programs. Thus, Baton Passing involves not only replacement of a molecular executor, but also recurrent recruitment of foreign viral genes into host biology.

This distinction is central to the Baton Pass model. In conventional antagonistic coevolution, molecular change often occurs through recurrent mutations within the same interacting genes. In non-orthologous gene displacement, different genes can perform similar functions. In Baton Passing, however, a host-level function can be maintained through successive domestication and replacement of genes of distinct viral origins. This feature makes the model particularly relevant to ERV-derived systems such as syncytins.

## 5. Mechanistic Drivers of Functional Instability

### 5.1. Receptor Conflict and Env–Receptor Instability

Retroviral Env proteins mediate membrane fusion through interactions with host cell surface receptors. These receptors are not evolutionarily static. Because many of them are exploited by exogenous viruses as entry receptors, they can be shaped by selective pressures that favor reduced viral susceptibility [30,31]. At the same time, endogenous retroviral Env proteins that have been domesticated for host functions may depend on the same or related receptor interfaces. This creates a form of evolutionary tension, here referred to as receptor conflict.

In this framework, Env–receptor compatibility is inherently unstable. Mutations or regulatory changes in host receptors may reduce susceptibility to exogenous viruses, but such changes can also compromise the activity of endogenized Env-derived fusogens. Conversely, viral Env proteins may evolve to regain receptor usage. Thus, the same molecular interface can be influenced by host defense, viral entry, and domesticated host function.

**Figure 3:**
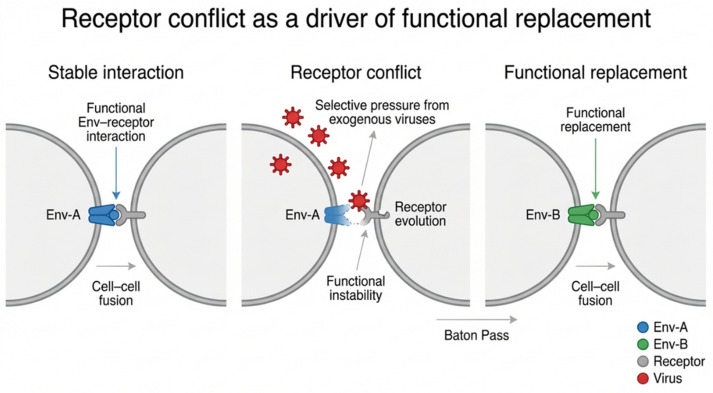
Receptor conflict as a driver of functional replacement. An Env-derived fusogen initially interacts efficiently with a host receptor to mediate cell–cell fusion. Under selection imposed by exogenous viruses that use the same or related receptor, receptor sequence or expression may change, reducing compatibility with the original Env protein. Recruitment of another Env-derived gene with a compatible receptor interaction can restore the host-level function. This process illustrates how receptor conflict can contribute to functional replacement in the Baton Pass model.

Receptor conflict provides one mechanistic explanation for why individual syncytin-like Env genes may have limited evolutionary lifespans. If receptor sequence, expression, or availability changes over time, a previously functional Env–receptor combination may become attenuated. Under such conditions, maintenance of the host-level function may favor recruitment of another Env-derived gene with a compatible receptor interaction. This process links host–virus co-evolution to functional replacement in the Baton Pass model (Figure 3).

### 5.2. Host–Virus Co-Evolution and Functional Replacement

Several retroviral receptor systems illustrate why Env–receptor interfaces are evolutionarily unstable. Sodium-dependent neutral amino acid transporters, including ASCT1 and ASCT2, serve as receptors for multiple retroviruses and are also used by several mammalian syncytins [8,13,32,33]. Comparative and functional studies of RD-114-related retroviruses and related Env proteins indicate that relatively small differences in extracellular receptor regions can markedly alter Env-mediated entry or fusion [32,33,34]. Such sensitivity supports the idea that receptor compatibility can be repeatedly gained, weakened, or lost during evolution.

Recent studies of the syncytin-1–ASCT2 interaction further illustrate this point [35,36,37]. Mutational and structural analyses have shown that receptor usage and fusogenic activity can be highly sensitive to specific residues within the Env–receptor interface. Such findings support the view that even limited molecular changes can alter the compatibility of a domesticated Env protein with its receptor, thereby contributing to the evolutionary instability of syncytin-like systems.

A similar principle is seen in other receptor systems. The sodium-dependent phosphate transporter PiT1/SLC20A1 serves as an entry receptor for multiple exogenous gammaretroviruses and is also used by murine syncytin-B [38]. These examples show that placental fusogens can retain molecular interfaces that overlap with those shaped by exogenous retroviral infection. The host may therefore face opposing pressures: to maintain receptor functions required for physiology and placentation, while limiting exploitation by infectious viruses.

Importantly, receptor conflict should not be viewed as the sole driver of syncytin succession. As discussed above, successful domestication of an env gene also requires regulatory compatibility, including appropriate placental or trophoblast-specific expression and suppression of potentially deleterious expression elsewhere. The Baton Pass model therefore does not attribute functional replacement to receptor change alone. Rather, it proposes that repeated turnover can arise when receptor compatibility, regulatory compatibility, and the availability of alternative ERV-derived env genes intersect.

In this view, receptor conflict acts as one destabilizing force that can shorten the functional lifespan of individual Env–receptor combinations. Endogenous retroviral reservoirs provide alternative candidates, and host regulatory networks determine whether those candidates can be incorporated into placental function. Rather than requiring indefinite preservation of a single Env–receptor pair, the Baton Pass model allows functional continuity through replacement of the molecular executor by another Env gene of distinct evolutionary origin.

## 6. Viral-Side Baton Passing: Anti-BST-2 Activity in HIV and SIV

### 6.1. Reassignment of Anti-BST-2 Function Among Viral Genes

The evolutionary logic underlying the Baton Pass model is not restricted to host genomes. A comparable pattern can be observed on the viral side of host–virus co-evolution, particularly in primate lentiviruses. In these systems, antagonism of the host restriction factor BST-2/tetherin is a conserved functional requirement, whereas the viral gene responsible for this activity differs among lineages. In this viral context, “function” refers to a virus-level requirement for efficient propagation, rather than a host-level biological function.

BST-2 restricts the release of enveloped viruses by tethering budding virions to the cell surface [39,40,41]. For HIV and SIV, overcoming BST-2-mediated restriction is important for efficient viral propagation. However, this function is not tied to a single conserved viral gene. In many SIV lineages, BST-2 antagonism is mediated primarily by Nef [39,42]. In other lineages, including HIV-2 and related viruses, Env can contribute to BST-2 antagonism. In pandemic HIV-1 group M, the major BST-2 antagonist is Vpu [40,43].

This reassignment is closely linked to species specificity. Viral factors that efficiently antagonize BST-2 in one host species may be ineffective against BST-2 from another species [44,45]. Cross-species transmission can therefore expose mismatches between viral antagonists and host restriction factors, creating selective pressure for alternative solutions. The acquisition of efficient Vpu-mediated antagonism in pandemic HIV-1 illustrates such functional reassignment. Similar functional plasticity is also observed outside primates; in feline immunodeficiency virus, the Env signal peptide has been identified as a potent BST-2 antagonist [46].

**Figure 4:**
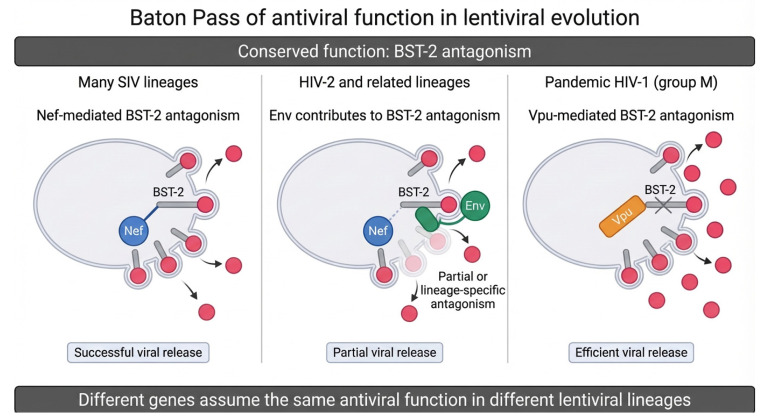
Viral-side Baton Passing of anti-BST-2 function in lentiviral evolution. The antiviral function—antagonism of BST-2/tetherin—is maintained, whereas the responsible viral factor changes among lineages. In different evolutionary contexts, Nef, Env, Vpu, or Env-derived peptide regions can counteract BST-2 and promote viral release. This example illustrates a viral-side Baton Pass, in which the same functional requirement is fulfilled by distinct genes or protein regions. Red circles indicate virions, and the X mark indicates antagonism of BST-2-mediated restriction by the indicated viral factor.

These examples provide a viral-side analogue of Baton Passing (Figure 4). The conserved function is antagonism of BST-2, but the molecular executor shifts among distinct viral proteins, including Nef, Env, Vpu, and Env-derived peptide regions. This pattern supports the broader principle that selection can preserve a biological function while allowing turnover of the gene responsible for executing it.

### 6.2. Lentiviruses as a Time-Compressed Model of Baton Passing

The reassignment of anti-BST-2 function among lentiviral genes parallels the functional succession proposed for ERV-derived env genes in host genomes. In both cases, a biological function is preserved while the molecular agent responsible for that function changes. In host genomes, this process is inferred from long-term comparative patterns of syncytin gain, attenuation, and replacement. In lentiviruses, similar dynamics can occur over much shorter evolutionary timescales because of high mutation rates, short generation times, and strong selective pressure imposed by host restriction factors.

For this reason, lentiviruses may serve as time-compressed models for testing the Baton Pass model. Functional loss, partial compensation, and reassignment to an alternative viral gene can potentially be observed experimentally by manipulating host restriction factors or disabling known viral antagonists. Such systems may allow direct investigation of the conditions under which a function is transferred from one molecular executor to another.

Thus, the lentiviral BST-2 system does not merely provide an analogy. It offers an experimentally tractable model for examining functional relay under host–virus conflict. Insights from these rapidly evolving viral systems may help clarify how similar principles operate over much longer timescales in host genomes, including the evolution of ERV-derived syncytins.

## 7. Testability and Future Directions

The Baton Pass model is intended not only as a conceptual interpretation of past evolutionary events, but also as a testable framework. In host genomes, Baton Passing can be examined by integrating comparative genomics, functional assays, and regulatory analyses. The model predicts that related species may retain comparable host-level functions while relying on different ERV-derived env genes. It also predicts that individual env genes should show temporally limited signatures of constraint, followed by relaxation or decay after functional replacement. These predictions can be tested by comparing coding integrity, fusogenic activity, receptor usage, placental expression, and regulatory elements across closely related species.

Functional studies will be particularly important. Candidate env-derived genes should be evaluated not only for fusogenic activity, but also for receptor compatibility and placenta- or trophoblast-specific expression. Conversely, attenuated or disrupted env genes should be interpreted in the context of possible functional replacement by other env-derived candidates. Such analyses may help distinguish Baton Passing from simple redundancy, relaxed selection, or ordinary gene decay.

Rapidly evolving viral systems provide a complementary experimental approach. As discussed above, lentiviruses offer time-compressed models in which functional loss, partial compensation, and reassignment to alternative genes may be observed over experimentally accessible timescales. Manipulating host restriction factors, viral antagonists, or receptor usage could allow direct testing of the conditions under which a biological function is transferred from one molecular executor to another.

Future work should therefore focus on identifying complete functional modules rather than coding sequences alone. In the syncytin paradigm, such modules include an env coding sequence, a compatible receptor interaction, and regulatory elements that ensure appropriate placental expression. Demonstrating coordinated replacement of these components would provide strong support for the Baton Pass model and clarify how host genomes repeatedly incorporate viral-derived genes into essential biological functions.

## 8. Generalization and Evolutionary Implications

The Baton Pass model was developed to explain the evolutionary dynamics of ERV-derived env genes in placental cell fusion, but its implications may extend beyond syncytins. The model is most likely to apply to biological systems in which three conditions are met: the host-level function is important enough that its loss is strongly selected against; the molecular executor of that function depends on an interface subject to persistent evolutionary instability; and a reservoir of alternative molecular candidates is available for recruitment.

ERV-derived env genes satisfy these conditions particularly well. Placental cell fusion is essential for normal development in many mammals, Env-mediated fusion depends on receptor interactions that can be shaped by host–virus conflict, and mammalian genomes contain numerous ERV-derived sequences that may serve as potential candidates for functional recruitment. In such systems, loss or attenuation of an individual env gene need not imply loss of the host-level function. Instead, it may reflect a transition in which the function has been reassigned to another viral-derived gene or to another functional module.

The model may also be relevant to other biological systems shaped by unstable molecular interfaces, including antiviral defense, cell–cell recognition, membrane fusion, and extracellular signaling. However, such comparisons require caution. Many immune-related gene families evolve through duplication, diversification, and positive selection, and these processes should not automatically be interpreted as Baton Passing. The key criterion is not merely gene turnover, but functional continuity through sequential replacement of the molecular executor.

This perspective also reframes the evolutionary role of endogenous retroviruses. ERVs are often viewed as genomic fossils or as occasional sources of evolutionary innovation [47,48,49]. The Baton Pass model suggests an additional role: ERVs can provide reservoirs of structurally related genes that allow host functions to persist despite instability of individual molecular solutions. Thus, recurrent loss of specific env genes is not necessarily paradoxical. It may be part of a broader evolutionary process in which host genomes repeatedly recruit, regulate, and replace viral-derived elements to maintain essential functions.

## 9. Conclusions

The Baton Pass model provides a framework for understanding how host-level biological functions can be maintained despite turnover of the genes that execute them. In the syncytin paradigm, trophoblast fusion is preserved across mammalian lineages even though the ERV-derived env genes responsible for this function differ in origin, integrity, activity, and regulation. This pattern suggests that functional continuity does not always require long-term conservation of a single molecular executor.

The model should be understood within the broader context of evolutionary conflict. The Red Queen hypothesis and antagonistic coevolution explain why molecular interfaces such as Env–receptor interactions may remain unstable. The Baton Pass model addresses a complementary question: how a host-level function can persist when the molecular executor itself is replaced. In this respect, Baton Passing differs from conventional examples of recurrent mutation within the same interacting genes and from ordinary non-orthologous gene displacement. Its distinctive feature is the recurrent acquisition, endogenization, domestication, and developmental integration of retroviral genes of different origins.

Successful Baton Passing requires more than replacement of coding sequences. In the case of syncytins, a functional relay must involve fusogenic activity, receptor compatibility, and regulatory compatibility, including appropriate placental or trophoblast-specific expression. Thus, the unit that is passed during evolution is best viewed as a functional module rather than a protein-coding gene alone.

By connecting comparative genomic evidence from ERV-derived env genes with experimentally tractable examples of viral functional reassignment, the Baton Pass model offers a testable evolutionary principle. It reframes gene loss not necessarily as loss of function, but potentially as part of a broader process of functional succession. More generally, this model suggests that biological continuity can be achieved not only through molecular conservation, but also through regulated replacement of the molecules that carry essential functions.

## Data Availability

No new data were created or analyzed in this study. Data sharing is not applicable to this article.

## References

[1] Crespi B., Semeniuk C. (2004). Parent-offspring conflict in the evolution of vertebrate reproductive mode. Am. Nat..

[2] Furness A.I., Morrison K.R., Orr T.J., Arendt J.D., Reznick D.N. (2015). Reproductive mode and the shifting arenas of evolutionary conflict. Ann. N. Y. Acad. Sci..

[3] Queller D.C., Strassmann J.E. (2018). Evolutionary conflict. Annu. Rev. Ecol. Evol. Syst..

[4] Imakawa K., Nakagawa S., Miyazawa T. (2015). Baton pass hypothesis: Successive incorporation of unconserved endogenous retroviral genes for placentation during mammalian evolution. Genes Cells.

[5] Imakawa K., Kusama K., Kaneko-Ishino T., Nakagawa S., Kitao K., Miyazawa T., Ishino F. (2022). Endogenous retroviruses and placental evolution, development, and diversity. Cells.

[6] Blond J.L., Lavillette D., Cheynet V., Bouton O., Oriol G., Chapel-Fernandes S., Mandrand B., Mallet F., Cosset F.L. (2000). An envelope glycoprotein of the human endogenous retrovirus HERV-W is expressed in the human placenta. J. Virol..

[7] Cornelis G., Heidmann O., Bernard-Stoecklin S., Degrelle S.A., Vernochet C., Lavialle C., Letzelter C., Hassanin A., Mulot B., Guillomot M. (2013). Captured retroviral envelope syncytin gene associated with the unique placental structure of higher ruminants. Proc. Natl. Acad. Sci. USA.

[8] Mi S., Lee X., Li X., Veldman G.M., Finnerty H., Racie L., LaVallie E., Tang X.-Y., Edouard P., Howes S. (2000). Syncytin is a captive retroviral envelope protein involved in human placental morphogenesis. Nature.

[9] Nakaya Y., Koshi K., Nakagawa S., Hashizume K., Miyazawa T. (2013). Fematrin-1 is involved in fetomaternal cell-to-cell fusion in Bovinae placenta and has contributed to diversity of ruminant placentation. J. Virol..

[10] Blaise S., de Parseval N., Bénit L., Heidmann T. (2003). Genomewide screening for fusogenic human endogenous retrovirus envelopes identifies syncytin-2. Proc. Natl. Acad. Sci. USA.

[11] Cornelis G., Vernochet C., Carradec Q., Souquere S., Mulot B., Catzeflis F., Nilsson M.A., Menzies B.R., Renfree M.B., Pierron G. (2015). Retroviral envelope gene captures and syncytin exaptation for placentation in marsupials. Proc. Natl. Acad. Sci. USA.

[12] Dupressoir A., Marceau G., Vernochet C., Benit L., Kanellopoulos C., Sapin V., Heidmann T. (2005). Syncytin-A and syncytin-B, two fusogenic placenta-specific murine envelope genes. Proc. Natl. Acad. Sci. USA.

[13] Mangeney M., Renard M., Schlecht-Louf G., Bouallaga I., Heidmann O., Letzelter C., Richaud A., Ducos B., Heidmann T. (2007). Placental syncytins: Genetic disjunction between the fusogenic and immunosuppressive activity of retroviral envelope proteins. Proc. Natl. Acad. Sci. USA.

[14] Shoji H., Kitao K., Miyazawa T., Nakagawa S. (2023). Potentially reduced fusogenicity of syncytin-2 in New World monkeys. FEBS Open Bio.

[15] Durnaoglu S., Lee S.K., Ahnn J. (2022). Syncytin, envelope protein of human endogenous retrovirus (HERV): No longer ’fossil’ in human genome. Anim. Cells Syst..

[16] Shimode S. (2023). Acquisition and exaptation of endogenous retroviruses in mammalian placenta. Biomolecules.

[17] Johnson W.E. (2019). Origins and evolutionary consequences of ancient endogenous retroviruses. Nat. Rev. Microbiol..

[18] Kitao K., Miyazawa T., Nakagawa S. (2022). Monotreme-specific conserved putative proteins derived from retroviral reverse transcriptase. Virus Evol..

[19] Kitao K., Shoji H., Miyazawa T., Nakagawa S. (2023). Dynamic evolution of retroviral envelope genes in egg-laying mammalian genomes. Mol. Biol. Evol..

[20] Nakaya Y., Miyazawa T. (2015). The roles of syncytin-like proteins in ruminant placentation. Viruses.

[21] Sakurai T., Kusama K., Imakawa K. (2023). Progressive exaptation of endogenous retroviruses in placental evolution in cattle. Biomolecules.

[22] Kitao K., Nakagawa S., Miyazawa T. (2021). An ancient retroviral RNA element hidden in mammalian genomes and its involvement in co-opted retroviral gene regulation. Retrovirology.

[23] Frost J.M., Amante S.M., Okae H., Jones E.M., Ashley B., Lewis R.M., Cleal J.K., Caley M.P., Arima T., Maffucci T. (2023). Regulation of human trophoblast gene expression by endogenous retroviruses. Nat. Struct. Mol. Biol..

[24] Yu M., Hu X., Pan Z., Du C., Jiang J., Zheng W., Cai H., Wang Y., Deng W., Wang H. (2023). Endogenous retrovirus-derived enhancers confer the transcriptional regulation of human trophoblast syncytialization. Nucleic Acids Res..

[25] Gimenez J., Montgiraud C., Oriol G., Pichon J.-P., Ruel K., Tsatsaris V., Gerbaud P., Frendo J.-L., Evain-Brion D., Mallet F. (2009). Comparative methylation of ERVWE1/syncytin-1 and other human endogenous retrovirus LTRs in placenta tissues. DNA Res..

[26] Ruebner M., Strissel P.L., Ekici A.B., Stiegler E., Dammer U., Goecke T.W., Faschingbauer F., Fahlbusch F.B., Beckmann M.W., Strick R. (2013). Reduced Syncytin-1 expression levels in placental syndromes correlates with epigenetic hypermethylation of the ERVW-1 promoter region. PLoS ONE.

[27] Sugimoto J., Schust D.J., Nagamatsu T., Jinno Y., Kudo Y. (2025). Genetic diversity in the suppressyn gene sequence: From polymorphisms to loss-of-function mutations. Biomolecules.

[28] Sugimoto J., Schust D.J., Sugimoto M., Jinno Y., Kudo Y. (2023). Controlling trophoblast cell fusion in the human placenta-transcriptional regulation of suppressyn, an endogenous inhibitor of syncytin-1. Biomolecules.

[29] Koonin E.V., Mushegian A.R., Bork P. (1996). Non-orthologous gene displacement. Trends Genet..

[30] Daugherty M.D., Malik H.S. (2012). Rules of engagement: Molecular insights from host–virus arms races. Annu Rev. Genet..

[31] Sironi M., Cagliani R., Forni D., Clerici M. (2015). Host-pathogen interactions and evolutionary signatures. Nat. Rev. Genet..

[32] Shimode S., Nakaoka R., Shogen H., Miyazawa T. (2013). Characterization of feline ASCT1 and ASCT2 as RD-114 virus receptor. J. Gen. Virol..

[33] Yoshikawa R., Yasuda J., Kobayashi T., Miyazawa T. (2012). Canine ASCT1 and ASCT2 are functional receptors for RD-114 virus in dogs. J. Gen. Virol..

[34] Miyaho R.N., Nakagawa S., Hashimoto-Gotoh A., Nakaya Y., Shimode S., Sakaguchi S., Yoshikawa R., Takahashi M.U., Miyazawa T. (2015). Susceptibility of domestic animals to a pseudotype virus bearing RD-114 virus envelope protein. Gene.

[35] Khare S., Villalba M.I., Canul-Tec J.C., Cajiao A.B., Kumar A., Backovic M., Rey F.A., Pardon E., Steyaert J., Perez C. (2024). Receptor-recognition and antiviral mechanisms of retrovirus-derived human proteins. Nat. Struct. Mol. Biol..

[36] Štafl K., Trávníček M., Kučerová D., Pecnová Ľ., Krchlíková V., Gáliková E., Stepanets V., Hejnar J., Trejbalová K. (2021). Heterologous avian system for quantitative analysis of Syncytin-1 interaction with ASCT2 receptor. Retrovirology.

[37] Štafl K., Trávníček M., Janovská A., Kučerová D., Pecnová Ľ., Yang Z., Stepanec V., Jech L., Salker M.S., Hejnar J. (2024). Receptor usage of Syncytin-1: ASCT2, but not ASCT1, is a functional receptor and effector of cell fusion in the human placenta. Proc. Natl. Acad. Sci. USA.

[38] Mousseau G., Préault N., Souquere S., Bireau C., Cassonnet P., Bacquin A., Beck L., Pierron G., Jacob Y., Dupressoir A. (2024). Sodium-dependent phosphate transporter PiT1/SLC20A1 as the receptor for the endogenous retroviral envelope syncytin-B involved in mouse placenta formation. J. Virol..

[39] Jia B., Serra-Moreno R., Neidermyer W., Rahmberg A., Mackey J., Ben Fofana I., Johnson W.E., Westmoreland S., Evans D.T. (2009). Species-specific activity of SIV Nef and HIV-1 Vpu in overcoming restriction by tetherin/BST2. PLoS Pathog..

[40] Neil S.J.D., Zang T., Bieniasz P.D. (2008). Tetherin inhibits retrovirus release and is antagonized by HIV-1 Vpu. Nature.

[41] Van Damme N., Goff D., Katsura C., Jorgenson R.L., Mitchell R., Johnson M.C., Stephens E.B., Guatelli J. (2008). The interferon-induced protein BST-2 restricts HIV-1 release and is downregulated from the cell surface by the viral Vpu protein. Cell Host Microbe.

[42] Lim E.S., Malik H.S., Emerman M. (2010). Ancient adaptive evolution of tetherin shaped the functions of Vpu and Nef in human immunodeficiency virus and primate lentiviruses. J. Virol..

[43] Sauter D., Schindler M., Specht A., Landford W.N., Münch J., Kim K.-A., Votteler J., Schubert U., Bibollet-Ruche F., Keele B.F. (2009). Tetherin-driven adaptation of Vpu and Nef function and the evolution of pandemic and nonpandemic HIV-1 strains. Cell Host Microbe.

[44] Yao X., Strebel K., Yamaoka S., Yoshida T. (2022). Simian immunodeficiency virus SIVgsn-99CM71 Vpu employs different amino acids to antagonize human and greater spot-nosed monkey BST-2. J. Virol..

[45] Yao X., Yoshida T., Hashimoto S., Takeuchi H., Strebel K., Yamaoka S. (2020). Vpu of a simian immunodeficiency virus isolated from greater spot-nosed monkey antagonizes human BST-2 via two AxxxxxxxW motifs. J. Virol..

[46] Morrison J.H., Poeschla E.M. (2023). The feline immunodeficiency virus envelope signal peptide is a tetherin antagonizing protein. mBio.

[47] Chuong E., Elde N., Feschotte C. (2017). Regulatory activities of transposable elements: From conflicts to benefits. Nat. Rev. Genet..

[48] Feschotte C., Gilbert C. (2012). Endogenous viruses: Insights into viral evolution and impact on host biology. Nat. Rev. Genet..

[49] Jiang R., Zhou J., Liu Y., Zhou G., Fan D., Xiang L., Chen Y., Shao J. (2025). Endogenous retroviruses in host-virus coevolution: From genomic domestication to functional innovation. Genes.

